# Enhanced Solubility and Rapid Delivery of Vitamin D3 via Captisol® (β-Cyclodextrin Sulfobutyl Ether) Inclusion Complex in Mouth-Dissolving Films

**DOI:** 10.1155/adpp/7621311

**Published:** 2025-08-18

**Authors:** Sultana Essa Bin Haider, Sharav Desai, Aliasgar F. Shahiwala

**Affiliations:** ^1^Department of Pharmaceutical Sciences, College of Pharmacy, Dubai Medical University, Dubai, UAE; ^2^Department of Pharmaceutical Biotechnology, Sanjivani College of Pharmaceutical Education & Research, Savitribai Phule Pune University, Kopargaon, Maharashtra, India

**Keywords:** captisol®, inclusion complex, mouth-dissolving films, vitamin D3

## Abstract

**Purpose:** Vitamin D3 deficiency is a global health concern affecting millions. However, its lipophilicity and poor water solubility limit its pharmaceutical applications. This study aims to improve the solubility of vitamin D3 by forming an inclusion complex with Captisol® (β-cyclodextrin sulfobutyl ether sodium salt) and developing a mouth-dissolving film (MDF) for its delivery.

**Methods:** The inclusion complex was prepared using a green, microwave-assisted method and characterized via molecular docking, phase-solubility studies, drug loading, and FTIR spectroscopy. The optimized complex was incorporated into MDFs which was further evaluated for their physicochemical and mechanical properties and sensory aspects.

**Results:** Molecular docking confirmed a high binding affinity (−10.7 K·mol^−1^) between vitamin D3 and Captisol®. The optimum stoichiometric ratio of vitamin D3 and Captisol® was 2:1 confirmed by the solubility curve, drug loading, and FTIR. The complex was incorporated into MDFs prepared using polyvinyl alcohol as a film former and Tween 80 as a plasticizer. The optimized films demonstrated desirable physicochemical and mechanical properties, rapid disintegration (36 s) and dissolution (complete release within the first 5 min), and excellent content uniformity (98.8%). Sensory evaluation revealed high acceptability, although improvements in the film's color and flavor were suggested.

**Conclusion:** This study establishes a novel, eco-friendly approach to enhance the solubility and patient-friendly delivery of vitamin D3.

**Summary**

• This study presents an innovative, eco-friendly method to enhance the solubility of vitamin D3 by forming a complex with Captisol® and delivering it via mouth-dissolving films (MDFs).
• The optimized films demonstrated rapid disintegration and dissolution, uniform drug content, and high patient acceptability, making them a convenient and effective option for vitamin D3 supplementation.
• The findings highlight a promising approach to addressvitamin D3 deficiency with improved pharmaceutical applications.

## 1. Introduction

Vitamin D3 is fundamental to calcium and phosphorus metabolism within the human body and is critical for the formation and maintenance of bone health, endocrine functions, and various other physiological systems. Recent research has expanded our understanding of vitamin D3's role in preventing a wide array of conditions, including cancer, cardiovascular diseases, diabetes, regulation of cellular proliferation and differentiation, embryonic development, fertility issues, immunological disorders, hepatic diseases, as well as neurological, renal, and respiratory system disorders [1–6]. However, the majority of the population throughout the globe is facing vitamin D3 deficiency, which is widely recognized as a global health concern [7]. Recently, the demand for vitamin D3 has increased, and it is widely used in food additives, pharmaceutical preparations, and feed additives [8].

Vitamin D3 deficiency affects millions of preschool-aged children [9]. Dietary intake alone often fails to meet the necessary vitamin D3 requirements, as the vitamin rapidly degrades during food processing and storage due to environmental stressors such as temperature, pH, salt, oxygen, and light. Consequently, the additional fortification of foods with vitamins becomes essential [10]. The lipophilic nature and poor water solubility of vitamin D3 (less than 1 mg per 100 g) also present challenges in its incorporation into pharmaceutical dosage forms and technological processes.

Despite extensive research on vitamin D3 and its critical role in human health, its lipophilicity and poor water solubility continue to limit its effective incorporation into pharmaceutical and nutraceutical products. While cyclodextrins (CDs), including β-CD and its derivatives, have been explored as solubilizing agents, most studies have focused on traditional CD derivatives rather than advanced modifications like Captisol®, which offers superior solubility, biocompatibility, and safety profiles [11, 12]. Furthermore, conventional methods of inclusion complex formation, such as codeposition and spray-drying, are often time-consuming, solvent-intensive, and less sustainable [13, 14].

Additionally, mouth-dissolving films (MDFs) have emerged as a promising drug delivery system due to their convenience, patient compliance, and rapid disintegration properties [15]. However, limited research has been conducted on the incorporation of CD-based complexes into MDF formulations. Existing studies on MDFs primarily focus on hydrophilic drugs or polymers with low mechanical challenges, leaving a gap in the development of films suitable for crystalline or lipophilic inclusion complexes like vitamin D3: Captisol® [16, 17].

To date, no study has systematically explored the molecular docking-guided formulation of a Captisol®:vitamin D3 inclusion complex, followed by its incorporation into a sustainable and patient-friendly MDF platform using a green synthesis technique. This research aims to address this critical gap by developing and characterizing a novel vitamin D3:Captisol® MDF formulation, thereby advancing the field of solubility enhancement and patient-centric drug delivery.

## 2. Materials

Captisol® was gifted by Ligand Pharmaceuticals (lot no. NC-04A-150146, San Diego, United States of America). Vitamin D3 (cholecalciferol), ethanol (AR grade), polyvinyl alcohol (PVA) (Selvol PVOH 203), and Tween 80 (standard grade) were purchased from Alpha Chemika (lot no. D0108F, Mumbai, India), Merck KGaA (lot no. K50966383904, Darmstadt, Germany), Sekisui Manufacturers (Tokyo, Japan), and ICI Americas, Inc. (New Jersey, United States), respectively. Diet sugar (Sweet'N Low Calorie Sweetener) was purchased from the local market.

### 2.1. Molecular Docking Studies

The molecular structural data file for vitamin D3 was obtained from the PubChem Database (CID_5280795) [18]. Captisol® represents a proprietary structure provided by Ligand Pharmaceuticals. The molecular structures were imported into PyRx, a software developed by the Molecular Graphics Laboratory at The Scripps Research Institute in La Jolla, CA, USA [19]. Vitamin D3 was used as a ligand, while Captisol® was utilized as a macromolecule. The ligand was subjected to energy minimization using the universal force field conjugate gradient method for 200 steps [20]. Before docking, both the macromolecule and ligand were converted into PDBQT format. The AutoDock Vina algorithm was used to predict the binding affinity between ligands and receptors [21, 22]. The ligand–receptor interaction was visualized in 3D using PyMOL and Biovia Discovery Studio.

### 2.2. Preparation of Standard Calibration Curve

The stock solution was prepared with accurately weighed 10 mg of vitamin D3, and dissolved 20% v/v  ethanol, in water. Afterward, 0.2, 0.4, 0.6, 0.8, and 1 mL of the resulting solution were diluted to 5 mL with 20% v/v ethanol in water. The absorption maxima (λmax) were determined by scanning 10 μg/mL solution against the blank on a UV–Visible spectrophotometer (UV-1700, Shimadzu, Japan) between 200 and 400 nm. A calibration curve for the drug was obtained by measuring the absorbance at the λmax against the blank. The calibration curve was produced and subjected to linear regression.

### 2.3. Preparation of Solubility Diagram

Phase-solubility studies were performed according to the method reported by Higuchi and Connors [23]. Vitamin D3 in amounts exceeding its solubility (10 mg) was transferred to different test tubes containing 5 mL of an aqueous solution of Captisol® (molecular weight = 1451.29 g/mol) in various molar concentrations (5 mM, 10 mM, 20 mM, and 40 mM). The contents were sonicated for 30 min and kept overnight to reach equilibrium. The samples were filtered through a 0.22-μm membrane filter, suitably diluted, and analyzed spectrophotometrically for drug content at λmax using a spectrophotometer. The stability constants, Kc, were calculated from the straight-line portion of the phase-solubility diagram according to the Higuchi–Connors equation [24]:

(1) $$\begin{array}{c} \text{Kc}=\frac{\text{Slope}}{S_{0}\left(1-\text{Slope}\right)}. \end{array}$$

### 2.4. Preparation of Captisol® With Vitamin D3 Complex

Microwave irradiation method, which is eco-friendly, cost-effective, and green technology, was used to prepare Captisol® and Vitamin D3 complex [14, 25]. Vitamin D3 with an equimolar or double molar quantity of Captisol® was ground in a glass container. Five mL of solvent (ethanol/water = 1:1 v/v) was added. The mixture was reacted for 90 s in the microwave oven (GMO1899, Geepas, Dubai, UAE) at low power settings. After the reaction, the solvents were dried at 40°C on a hot plate, and samples were stored in amber-colored airtight containers in a refrigerator for further characterization.

### 2.5. Determination of Entrapped Vitamin D3

The weighted (10 mg) dried complex was dissolved in the required amount of water, followed by centrifugation at 5000 rpm for 10 min to separate unentrapped vitamin D3. Samples were visually observed to check for vitamin D3. Samples were filtered through 0.22-μm syringe filters, and entrapped vitamin D3 was determined spectrophotometrically.

### 2.6. FTIR Spectroscopy Analysis of Captisol®/Vitamin D3 Complex

Using PerkinElmer 100 FTIR with an attenuated total reflectance (ATR) accessory (resolution of 3.1 microns) (PerkinElmer, Inc. Massachusetts, USA), an IR spectroscopy in the 200–4000 cm^−1^ wavenumber range was performed at room temperature to investigate Captisol®, vitamin D3, and the 2:1 Captisol®:vitamin D3 complex.

### 2.7. Preparation of MDFs Containing Vitamin D3 and Captisol® Complex

MDFs were prepared using the casting method (Mashru RC, 2005). Nine different formulations of MDF were attempted, as shown in Table 1. PVA was soaked in water for 1 hour and then uniformly dispersed to obtain dispersion. Other formulation components, including 2:1 Captisol®:vitamin D3 complex and a plasticizer, were added with continuous stirring with a glass rod for 15–20 min. The contents were subjected to sonication for 20 min. The aqueous dispersion was then refrigerated for 6 h to settle the foam formed due to stirring. The aqueous dispersion was cast onto a glass petri dish and dried at 60°C overnight in the oven. The films were evaluated for their proper formation, ease of removal from the petri dish, and brittleness to obtain the optimized formulation. The required amount of sweetener was added to the optimized formula, and optimized films were cut into squares 2 cm × 2 cm for further characterizations.

### 2.8. Evaluation and Characterization of MDF

#### 2.8.1. Tackiness

Three films from the final formula were chosen at random. The tackiness of each strip was measured by pressing it against the fingertips [26].

#### 2.8.2. Thickness

The thickness of the film was measured using a digital vernier caliper for three films.

#### 2.8.3. Weight Variation

Three films were individually weighed by an analytical balance. The mean weight (in milligrams) with the standard deviation (SD) was calculated [15].

#### 2.8.4. Folding Endurance

Fold endurance is a metric that determines how often a film can be folded before it breaks when subjected to steady stress [27]. The film was folded at the same place to determine the number of times it folded before breaking.

#### 2.8.5. Assay and Content Uniformity

An accurately weighed portion of the film (about 20 mg) was dissolved in 10 mL of 20% ethanol to determine the drug content uniformity and assay. Drug content was determined using a developed calibration curve after appropriate dilution. This test was repeated for three different films.

#### 2.8.6. pH

The pH of the film was determined after dissolving the film in 100 mL double distilled water using a digital pH meter (Hanna Instruments Inc., HI 8417, Woonsocket, USA).

#### 2.8.7. Tensile Strength and Elongation

**Figure 1:**
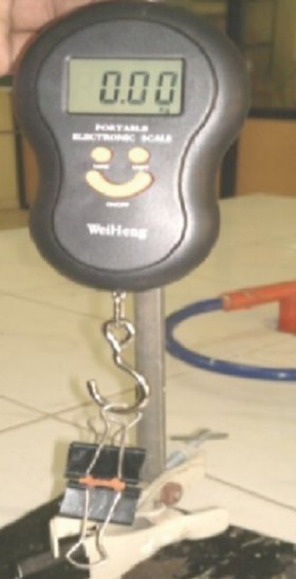
Setup for measurement of tensile strength of the film.

Tensile strength refers to the maximum stress film can withstand [15, 28].  Figure 1 shows a portable scale (WeiHeng portable digital scale, Anglers, WH-22, Dubai, UAE) with two clamps. The film was held between the two clamps, and weights were applied until the strip broke. The tensile strength was then calculated by the applied load at rupture divided by the cross-sectional area of the film as given in the equation below:

(2) $$\begin{array}{c} \text{Tensile strength}=\frac{\text{Force at breakage }\left(\text{kg}\right)}{\text{Film thickness }\left(\text{mm}\right)\times \text{film width }\left(\text{mm}\right)}. \end{array}$$

Percent elongation was measured by pulling the film apart till it broke, and an increase in the length of the film at break was measured using a scale. % Elongation is calculated by the formula:

(3) $$\begin{array}{c} \%\text{ Elongation}=\frac{\text{Increase in length of strip}\times 100}{\text{Initial length of strip}}. \end{array}$$

#### 2.8.8. Disintegration Test

The disintegration test aims to ensure that the pill will disintegrate over time. Three films were carefully placed on the surface of a petri dish filled with 2 mL of distilled water. The time it took for the films to disintegrate completely was recorded [28].

#### 2.8.9. Dissolution Test

The in vitro dissolution profile of the MDFs containing the Captisol®–vitamin D3 inclusion complex was evaluated using USP Apparatus II (paddle method). The dissolution studies were performed in 250 mL of dissolution medium maintained at 37 ± 0.5°C with a paddle rotation speed of 50 rpm. Based on the poor aqueous solubility of vitamin D3, a dissolution medium consisting of distilled water containing 1.2% (w/v) sodium lauryl sulfate (SLS) was selected to ensure sink conditions, in accordance with USP < 711 > guidelines and published literature [29, 30].

Film strips equivalent to of vitamin D3 were carefully placed in the dissolution vessels. At predetermined time intervals (5, 10, 15, 30, 45, and 60 min), 5 mL aliquots were withdrawn and replaced with fresh prewarmed medium to maintain a constant volume. The samples were filtered through a 0.45-μm nylon syringe filter and analyzed for vitamin D3 content using a UV spectrophotometer [31]. All experiments were conducted in triplicate, and cumulative drug release was plotted as a function of time.

### 2.9. Sensory Evaluation

A sensory panel of 12 human volunteers (six males and six females) provided with an informed consent form describing information regarding the product, its quality-related terms, and its quality assessment procedure was recruited for the product sensory assessment. The Ethical Committee of Dubai Pharmacy College for Girls approved the human studies protocol (DPC-/REC/MPharm/2020/02). The volunteers were offered the minimum amount of the film (0.2 × 0.2 cm). Volunteers were asked to rate their perceptions using a Likert scale from 1 (poor) to 5 (excellent) on the sensory aspects of the film, like the taste, sweetness, dissolving time, appearance, shape, color, odor, and texture of the product.

### 2.10. Statistical Analysis

All quantitative data are expressed as mean ± SD, and statistical significance was determined using appropriate statistical tests, with significance explicitly reported as *p*-values. Results with a *p*-value less than 0.05 were considered statistically significant, and confidence intervals (95% CIs) were included where applicable to demonstrate the precision of our findings.

## 3. Results and Discussion

To improve the water solubility of vitamin D3, the complexation with CDs has been proposed [32–34]. CDs are cyclic oligosaccharides that have an inner hydrophobic cavity and a hydrophilic outer shell. This unique structure allows hydrophobic compounds to be entrapped within the internal cavity of the CDs, creating “guest–host” type inclusion complexes [35]. The inclusion complex offers various advantages, such as increasing the photo- and oxidation stability [36], improved solubility and bioavailability [37], masking unpleasant odor and taste [38], and converting liquid drugs into powder form [39].

Captisol®, a modified sulfobutyl ether, is a polyanionic derivative of beta-CD. Compared to beta-CD's solubility of 1.85 g/100 mL at 25°C [40], Captisol® demonstrates superior water solubility at 70 g/100 mL [41]. It is designed to enhance safety and optimize interactions with the substracts to improve their solubility, stability, and bioavailability while minimizing volatility, irritation, odor, and taste [12]. Furthermore, Captisol® exhibits no nephrotoxicity associated with β-CD [11] or cytotoxic effects on intestinal epithelial Caco-2 cells [42], so it can be safely administered orally. Many Captisol® added products are approved by the FDA or are in clinical trials [43].

### 3.1. Molecular Docking Studies

Traditional methods for studying inclusion complexation often rely solely on experimental techniques, which can be resource-intensive and time-consuming. Computational docking studiesprovides valuable insights into binding interactions and optimizes experimental design [44]. AutoDock Vina was employed for this purpose due to its enhanced computational efficiency and accuracy in predicting ligand–receptor interactions compared to other algorithms like AutoDock 4.2 [21]. The algorithm's optimized scoring function and multithreading capabilities allowed rapid exploration of the binding site while reliably estimating binding energies, making it suitable for hydrophobic compounds like vitamin D3. Previous studies have validated the robustness of Vina for complexation with CDs, further supporting its selection for this investigation [22, 45].

**Figure 2:**
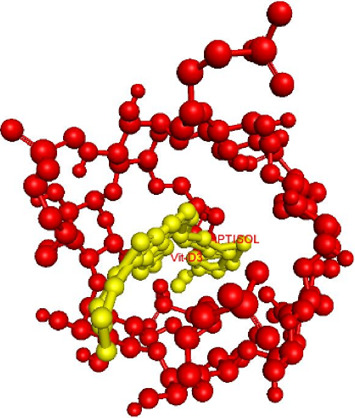
Optimal docking model of the inclusion complexation.

The docking results revealed a high binding affinity of −10.7 kcal/mol between vitamin D3 and Captisol® (Table 2), indicating a stable inclusion complex. This binding energy surpasses values reported for other CD complexes, such as β-CD or hydroxypropyl β-CD, in similar studies [45, 46]. The optimal docking model, visualized in PyMOL (Figure 2), confirmed that vitamin D3 was securely encapsulated within Captisol®'s hydrophobic cavity.

The results highlight the importance of molecular docking as a preliminary tool to predict complex stability and binding behavior before experimental validation. The negative docking energy obtained in this study suggests strong hydrophobic interactions between vitamin D3 and Captisol®, correlating well with the solubility enhancements observed experimentally. These findings align with earlier research demonstrating the effectiveness of CD derivatives in solubilizing hydrophobic drugs through inclusion complex formation [24].

### 3.2. Preparation of Captisol® With Vitamin D3 Complex

In pharmaceutical research, conventional methods such as kneading, coprecipitation, freeze-drying, and spray-drying have historically been employed for CD-based inclusion complex formation [47]. The freeze-drying and coprecipitation processes are also used to produce inclusion complexes [13, 48]. These conventional techniques often suffer from inherent limitations, including prolonged processing times, significant solvent consumption, elevated energy requirements, and limited scalability, which collectively affect the efficiency, economic feasibility, and environmental sustainability of the process [49, 50]. Conversely, the microwave-assisted synthesis employed in the present study has emerged as an attractive alternative due to its superior efficiency, marked by significantly reduced reaction times (minutes vs. hours) and energy consumption, lower solvent usage, resulting in lower production costs and enhanced scalability potential [33, 50, 51]. Microwave-assisted methods utilize rapid and uniform heating mechanisms that accelerate complexation reactions, making them particularly suitable for green chemistry principles by minimizing environmental impact and enhancing process sustainability [50]. This clear distinction highlights the rationale behind choosing microwave-assisted synthesis in our study, aligning it with modern, economically viable, and environmentally responsible pharmaceutical manufacturing practices.

### 3.3. Vitamin D3 Calibration Curve in Methanol:Water (20:80% v/v)

The calibration curve suggested a linear relation between drug concentration and absorbance. The curves obey Beer–Lambert's law within the concentration range of 2–10 μg/mL (*R*^2^ > 0.999) of vitamin D3 at λmax of 268.8 nm. The high value of *R*^2^ suggests a low prediction error of the equation and was used to determine the vitamin D3 solubility, assay, and entrapment.

### 3.4. Phase-Solubility Diagram

**Figure 3:**
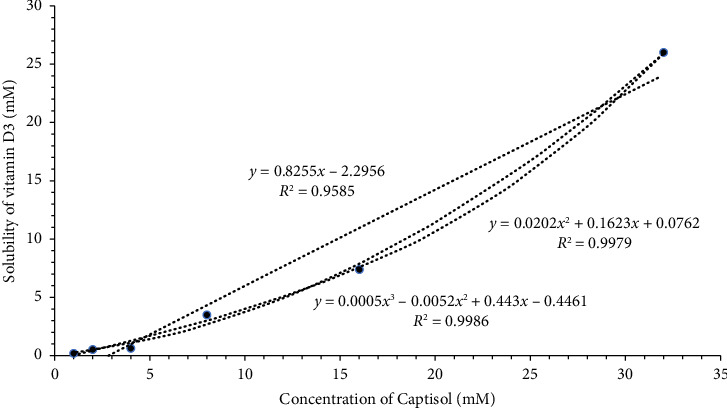
Solubility diagram showing a linear, quadratic, and cubic relationship between the concentration of Captisol® and solubility of vitamin D3.

The phase-solubility diagram assesses the solubilizing effect of complexing agents on the drugs, the stability constant, and the stoichiometry of the equilibrium [24]. Our solubility diagram suggests an Ap type of isotherm between Captisol® concentration and solubility of vitamin D3, suggesting a positive deviation from linearity (Figure 3). This indicates higher-order complexes concerning the Captisol (i.e., vitamin D3•2Captosol®, vitamin D3•3Captosol®); the Captisol® is proportionally more effective at higher concentrations. The stoichiometry of the formed complexes can be determined by the extent of curvature of the phase-solubility profile, which states that if an isotherm best fits a quadratic function, it suggests the formation of a one-to-two (vitamin D3•2Captosol®) complex and if it best fits a cubic function, it suggests a one-to-three complex (vitamin D3•3Captosol®), and so forth [23]. The *r*^2^ values of linear, quadratic, and cubic functions for our solubility diagram were found to be 0.9585, 0.9979, and 0.9986, respectively, which indicates a quadratic relationship for our complex formation; that is, one-to-two ((vitamin D3•2Captosol®) can sufficiently explain the stoichiometric ratio of our complex [17]. The theoretical stability constant calculated from the initial right portion of the curve is 2.78, which suggests sufficient stability of the complex [24].

The quadratic equation suggesting the formation of a 1:2 drug/CD complex [47]:

(4) $$\begin{array}{c} S_{\text{tot}}=S_{0}+K_{1:1}S_{0}\left[\text{CD}\right]+K_{1:1}K_{1:2}S_{0}{\left[\text{CD}\right]}^{2}. \end{array}$$

For our complex, the values of *S*_0_, *K*_1:1_*S*_0_, and *K*_1:1_*K*_1:2_*S*_0_ were found to be 0.0762, 0.1623, and 0.0202, respectively (Figure 3). While solving, the coefficient *K*_1:1_ and *K*_1:2_ values were found to be 2.13 and 0.124, respectively, suggesting the weaker interaction of the secondary complex compared to the primary complex of vitamin D3:Captisol®. In a similar study, the CD complex with pyrazoline antioxidants, similar to vitamin D3, also suggested a 1:2 drug:CD complex [52].

### 3.5. Determination of Entrapped Vitamin D3

The entrapped percentage of vitamin D3 was 99.1%, which indicates complete entrapment in a 2:1 ratio of Captisol®:vitamin D3.

### 3.6. FTIR Spectroscopy Analysis

**Figure 4:**
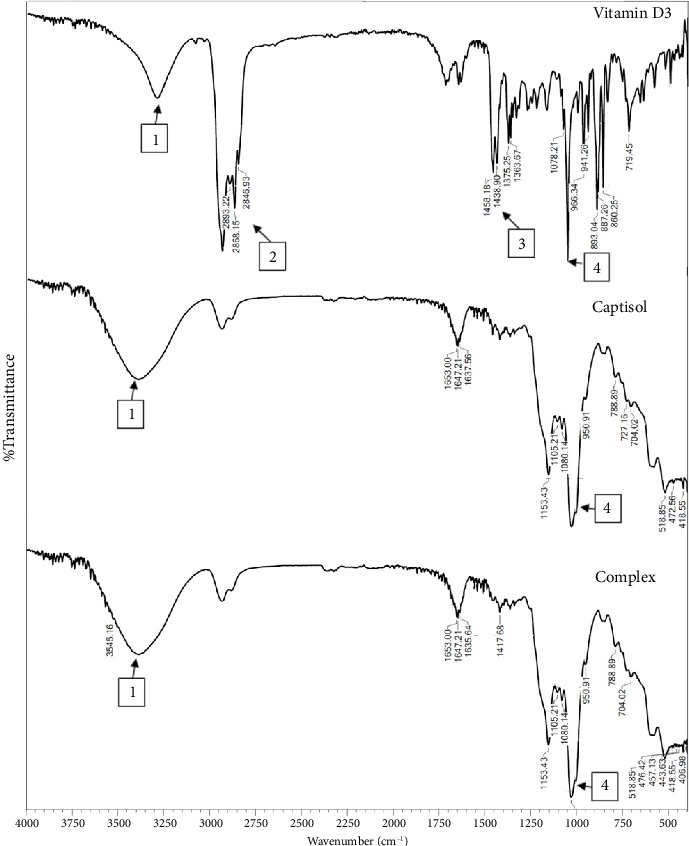
FTIR spectra of Captisol® (top), vitamin D3 (middle), and complex (bottom).

Fourier transform infrared was performed to detect the possible molecular interaction between Captisol® and vitamin D3 (Figure 4).

The characteristic absorption bands of vitamin D3 were O-H stretching for the alcoholic group. The carboxylic acid appears as broadband between ∼3600 cm^−1^ and 3100 cm^−1^ and centered at ∼3300 cm^−1^. A series of bands were observed in the spectrogram of vitamin D3, namely hydrogen bond O-H stretching (3289 cm^−1^) (1), alkyl C-H stretches (2893 cm^−1^ and 2868 cm^−1^, 2846 cm^−1^) (2), C=O stretching (1458 cm^−1^, 1438.9 cm^−1^) (3), C-O stretching (1078 cm^−1^) (4), CH2 group (719 cm^−1^), and several other picks in the wavenumber range of 860–966 cm^−1^ indicating C-H bending. The IR spectra of Captisol® showed strong absorption bands at 3423.46 cm^−1^ (O-H stretching) (1), 2937.51 cm^−1^ (C-H stretching) (2), 1647 cm^−1^ (δ-HOH bending of water molecules attached to CD), 1153 (C-H vibrations), and 1028 cm^−1^ (C-O stretching) (4), respectively [52]. However, in the spectra FTIR of the vitamin D3:Captisol® inclusion complex, most of the vitamin D3 picks are absent, suggesting the entrapment of vitamin D3 inside the Captisol [53].

### 3.7. Preparation and Optimization of MDFs

**Figure 5:**
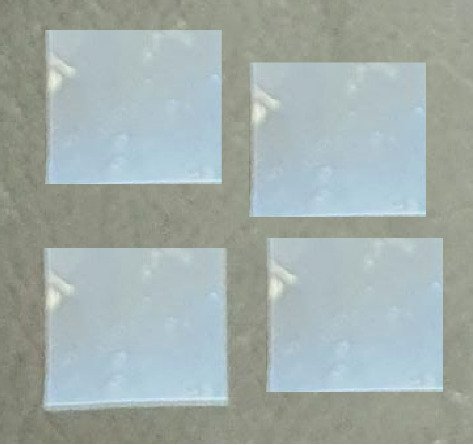
Digital photographs of the optimized Captisol®: vitamin D3 complex MDF (2 ∗ 2 cm^2^).

CDs have low viscoelasticity and low Young's modulus [54], necessitating the other polymer to impart suitable mechanical properties to the strips. Initial trials were taken with hydroxypropyl methyl cellulose (HPMC) polymers and propylene glycol (PG) as a plasticizer. The initial choice of HPMC as a film-forming polymer was guided by its widespread acceptance in oral film formulation, offering desirable flexibility and mechanical integrity. HPMC E5 and E15 in different ratios were used to optimize the blank film formulations (F1–F3), as these polymers are widely used as film formers in MDFs [16]. HPMC E5 showed better film-forming properties and was selected further with the trials. However, after incorporating the Captisol®:vitamin D3 complex in the formulations, HPMC E5 film properties were changed completely, resulting in brittle films. This may be attributed to the crystalline nature of the inclusion complex, which altered the polymer's mechanical properties, resulting in compromised structural integrity [17]. Consequently, we transitioned to PVA, selected due to its superior film-forming properties, water solubility, biodegradability, and compatibility with inclusion complexes [55, 56]. PVA successfully produced films (F8 and F9) that maintained the required flexibility, tensile strength, and structural uniformity, thereby addressing the brittleness issues encountered with HPMC. Additionally, PVA's compatibility with Tween 80 as a plasticizer further contributed to the enhanced ductility and handling characteristics of the optimized MDF formulation [57]. The formula F9 with 750 mg of PVA as a film former and 60 mg Tween 80 as a plasticizer was found to be optimum. In the same formula, 50 mg of commercial sweetener was added to impart sufficient sweetness to the film. The digital photograph of the film is shown in Figure 5.

### 3.8. Evaluation of Optimized MDF

The optimized MDF was characterized by different parameters, as listed in Table 3.

#### 3.8.1. Tackiness

For the tackiness of the films, it was observed that they were not sticky, and the appearance was colorless and transparent.

#### 3.8.2. Thickness

The film's thickness should be uniform, affecting the weight variation and the dose accuracy. As per an earlier report, the desirable thickness for an ideal oral film should be between 0.05 and 1 mm [58]. The average thickness of the films in this study was found to be 0.12 mm.

#### 3.8.3. Weight Variation

The average weight of 2 × 2 films was 19.8 mg with a weight variation of 8.6% (1.7 mg), within the limit as per USP, BP, and IP.

#### 3.8.4. Tensile Strength

An ideal MDF should have an appropriate tensile strength to withstand handling during manufacturing. It should not be very high, which indicates high rigidity, as it could retard the drug release from the polymer matrix [59]. The tensile strength of the prepared films was 1.2 MPa, which was appropriate [60].

#### 3.8.5. Folding Endurance and % Elongation

The elasticity and flexibility of the prepared MDF are important physical characteristics required for easy handling and the application of the MDF to the oral cavity [59]. Strain refers to the change in the shape of a material before it fails because of stress, quantified by the percentage of elongation [61]. Generally, the elongation of the film increases as the plasticizer content increases. Sufficiently high values for folding endurance (> 100) and % elongation (39.8 ± 2.7%) were obtained, which indicates that the desired films have suitable flexibility and the ability to withstand the strain. This is attributed to the presence of the plasticizer (Tween 80).

#### 3.8.6. pH

The pH of the film was found to be 5.9 ± 0.2, which is close to the salivary pH (6.2–7.6) [62] and hence less likely to cause any mucosal irritation.

#### 3.8.7. Assay and Content Uniformity

The assay results for drug content demonstrated excellent uniformity at 98.8 ± 2.1% (95% CI: 96.7%–100.9%), indicating robust and precise formulation characteristics that meet pharmacopeial standards (95%–105%).

#### 3.8.8. Disintegration

The disintegration time of the optimized MDF was found to be 36 ± 13 s, significantly shorter than conventional formulations (*p* < 0.05, 95% CI: 28–44 s), reflecting rapid and reliable performance. This enhances patient compliance, particularly for populations with swallowing difficulties, such as pediatric and geriatric patients. The disintegration time complies with USFDA guidelines, ensuring quick drug release in saliva for absorption via oral or gastrointestinal routes [63]. The inclusion of Captisol® improved the solubility of vitamin D3, while the use of PVA as a film-forming polymer and Tween 80 as a plasticizer facilitated rapid water uptake and matrix breakdown. These features ensure minimal lag between administration and therapeutic action, making the MDFs a patient-friendly and effective delivery system for vitamin D3 supplementation.

#### 3.8.9. Dissolution Studies

**Figure 6:**
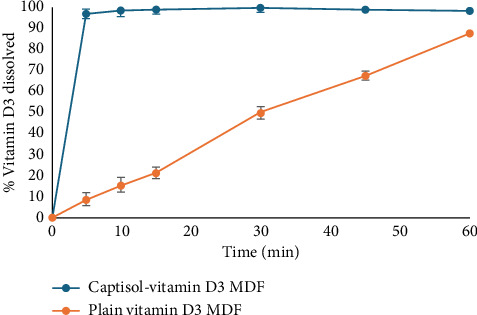
Comparative dissolution performance of Captisol®: vitamin D3 and plain vitamin D3 films.

The dissolution behavior of Captisol®-vitamin D3 inclusion complex formulated into MDFs was evaluated in comparison to a control film containing plain (uncomplexed) vitamin D3. As shown in Figure 6, the Captisol®-based MDF exhibited a significantly high (*p* < 0.05) and complete release, with over 96% of vitamin D3 released within the first 5 min, and reaching ∼99% release by 10 min. In contrast, the plain vitamin D3 MDF showed a gradual release, achieving only ∼22% release at 10 min, ∼51% at 30 min, and ∼88% by 60 min.

These results clearly indicate the superior dissolution performance of the CD-based formulation. The rapid release observed with the Captisol® complex can be attributed to the significant enhancement in aqueous solubility and dispersion provided by the sulfobutyl ether β-CD inclusion. Our findings are consistent with earlier studies highlighting the significance of CD-based solubilization strategies [29, 33].

### 3.9. Sensory Evaluation

**Figure 7:**
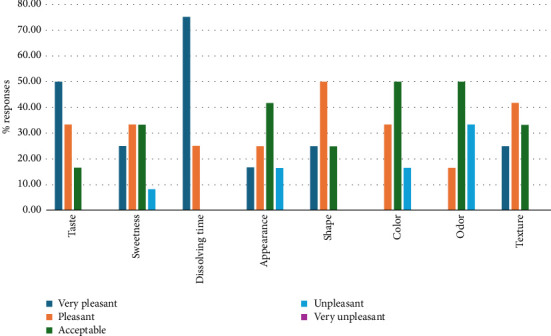
Sensory evaluation of vitamin D3 films.

A sensory panel of 12 human volunteers was recruited for the product's sensory evaluation. Each participant provided an informed consent form that included detailed information about the product, its quality-related parameters, and the procedures for quality assessment. Each volunteer was presented with a film, and their perceptions of the film's sensory attributes, such as taste, sweetness, dissolution time, appearance, shape, color, odor, and texture, were recorded. These sensory evaluations were conducted using an ordinal scale ranging from very pleasant (5) to very unpleasant (1). The volunteers showed high acceptance of the film, reflected in their pleasant to very pleasant responses for most of the sensory aspects (Figure 7). However, the film's color, odor, and appearance were acceptable and needed to be improved by incorporating color, odor, and flavor-enhancing agents. Sensory evaluation data were statistically analyzed using the Friedman test, a nonparametric method suitable for ordinal data from sensory panels. Significant differences (*p* < 0.05) among sensory attributes were identified, followed by post hoc Wilcoxon signed-rank tests with Bonferroni correction to pinpoint specific differences. 95% CIs for median scores were calculated to provide additional insights into the reliability of sensory acceptability ratings. Results revealed statistically significant higher scores for taste (median = 4.5, 95% CI: 4.0–5.0), dissolving time (median = 5.0, 95% CI: 4.5–5.0), and texture (median = 4.0, 95% CI: 3.5–4.5) compared to odor (median = 3.0, 95% CI: 2.5–3.5), significantly lower than taste and dissolving time (*p* < 0.05) and color (median = 3.5 (95% CI: 3.0–4.0), significantly lower than dissolving time (*p* < 0.05).

In addressing the limitations associated with existing vitamin D3 delivery methods, traditional dosage forms such as tablets, soft gels, and oral solutions predominantly suffer from low bioavailability due to the lipophilic nature and poor aqueous solubility of vitamin D3. Tablets and soft gels, although commonly used, encounter drawbacks including variable absorption, first-pass metabolism, and limited dissolution rates, particularly in populations with impaired gastrointestinal function or reduced gastric acidity [64]. In contrast, the developed MDFs incorporating vitamin D3 complexed with Captisol® markedly address these limitations by enhancing solubility and potentially improving bioavailability through rapid dissolution and absorption via the sublingual and buccal mucosa [12, 37]. The buccal and sublingual route offers distinct advantages by circumventing gastrointestinal and hepatic first-pass metabolism, potentially improving systemic bioavailability [65–68]. Although direct comparative bioavailability studies with commercially available formulations such as tablets and soft gels were beyond the scope of this study, the literature consistently indicates superior pharmacokinetic profiles for CD-based inclusion complexes compared to conventional dosage forms. Previous research on Captisol® demonstrates enhanced solubility, improved dissolution rates, and increased bioavailability for poorly soluble vitamins and other hydrophobic compounds [41, 46]. Captisol® inclusion complexes exhibited significantly higher dissolution rates and enhanced gastrointestinal absorption compared to traditional CD derivatives like hydroxypropyl beta-CD, establishing a solid theoretical and empirical basis for expecting superior performance of MDFs formulated in this study [11, 43]. Future comparative bioavailability and pharmacokinetic studies with commercially available vitamin D3 products will further substantiate these preliminary conclusions and conclusively establish MDFs as a superior vitamin D3 delivery method.

## 4. Conclusions

This work successfully demonstrates the preparation of a vitamin D3:Captisol® inclusion complex using a sustainable, microwave-assisted method, which requires minimal solvents and energy. The complex achieved a high binding affinity and an optimal stoichiometric ratio of 2:1, significantly improving the solubility of vitamin D3. Incorporation into MDFs further enhanced its suitability for patient administration by offering rapid disintegration and dissolution, acceptable mechanical properties, and excellent content uniformity. Sensory evaluation highlighted the high acceptability of the films, although minor enhancements in esthetic properties could further optimize patient compliance. Overall, this research paves the way for developing an innovative, patient-friendly vitamin D3 formulation with improved solubility and bioavailability, addressing the challenge of vitamin D deficiency.

## Figures and Tables

**Table 1:** Formulation trials of MDF containing 2:1 Captisol®: vitamin D3 complex.

Component	F1	F2	F3	F4	F5	F6	F7	F8	F9
2:1 Captisol®:vitamin D3	—	—	—	102.2 mg:15.4 mg	102.2 mg:15.4 mg	102.2 mg:15.4 mg	102.2 mg:15.4 mg	102.2 mg:15.4 mg	102.2 mg:15.4 mg
HPMC E5	100 mg	—	50 mg	200 mg	300 mg	500 mg	750 mg	—	—
HPMC E15	—	100 mg	50 mg	—	—	—	—	—	—
PVA	—	—	—	—	—	—	—	500 mg	750 mg
PG	25 mg	25 mg	25 mg	25 mg	25 mg	—	—	—	—
Tween 80	—	—	—	—	—	60 mg	60 mg	60 mg	60 mg
Water	5 mL	5 mL	5 mL	5 mL	5 mL	5 mL	5 mL	5 mL	5 mL

**Table 2:** Binding energy of vitamin D3 with Captisol®.

Ligand	Binding affinity	RMSD/ub	RMSD/lb
captisol_5280795_uff_E = 4916289914203362.00	−10.7	0	0
captisol_5280795_uff_E = 4916289914203362.00	−10.3	2.881	1.69
captisol_5280795_uff_E = 4916289914203362.00	−9.9	2.786	1.62
captisol_5280795_uff_E = 4916289914203362.00	−9.9	2.024	1.61
captisol_5280795_uff_E = 4916289914203362.00	−9.7	6.575	1.807
captisol_5280795_uff_E = 4916289914203362.00	−9.5	6.863	2.801
captisol_5280795_uff_E = 4916289914203362.00	−9.1	6.665	1.947
captisol_5280795_uff_E = 4916289914203362.00	−9	3.986	2.691
captisol_5280795_uff_E = 4916289914203362.00	−8.9	7.001	2.594

Abbreviations: RMSD = root mean square deviation; RMSD/lb = RMSD lower bound; RMSD/ub = RMSD upper bound.

**Table 3:** Evaluation of optimized mouth-dissolving film containing Captisol®: vitamin D3 complex.

Evaluation test	Results (mean ± SD)
Tackiness	Nontacky
Folding endurance	> 100
Thickness	0.12 mm ± 0.03 mm
Weight (2 × 2 strips)	19.8 mg ± 1.7 mg
Tensile strength	1.2 MPa
% Elongation	39.8 ± 2.7%
pH	5.9 ± 0.2
Assay	98.8 ± 2.1%
Disintegration time	36 ± 13 s
Dissolution time	< 5 min

## Data Availability

Data sharing is not applicable to this article as no new data were created or analyzed in this study.

## References

[1] Atteritano M., Mirarchi L., Venanzi-Rullo E. (2018). Vitamin D Status and the Relationship With Bone Fragility Fractures in HIV-Infected Patients: A Case Control Study. *International Journal of Molecular Sciences*.

[2] Wierzbicka J. M., Binek A., Ahrends T. (2015). Differential Antitumor Effects of Vitamin D Analogues on Colorectal Carcinoma in Culture. *International Journal of Oncology*.

[3] Slominski A. T., Brożyna A. A., Skobowiat C. (2018). On the Role of Classical and Novel Forms of Vitamin D in Melanoma Progression and Management. *The Journal of Steroid Biochemistry and Molecular Biology*.

[4] Legarth C., Grimm D., Wehland M., Bauer J., Krüger M. (2018). The Impact of Vitamin D in the Treatment of Essential Hypertension. *International Journal of Molecular Sciences*.

[5] Abu el Maaty M. A., Almouhanna F., Wölfl S. (2018). Expression of TXNIP in Cancer Cells and Regulation by 1,25(OH)_2_D_3_: Is It Really the Vitamin D3 Upregulated Protein?. *International Journal of Molecular Sciences*.

[6] Maurya V. K., Bashir K., Aggarwal M. (2020). Vitamin D Microencapsulation and Fortification: Trends and Technologies. *The Journal of Steroid Biochemistry and Molecular Biology*.

[7] Holick M. F., Chen T. C. (2008). Vitamin D Deficiency: A Worldwide Problem With Health Consequences. *The American Journal of Clinical Nutrition*.

[8] Holick M. F. (2017). The Vitamin D Deficiency Pandemic: Approaches for Diagnosis, Treatment and Prevention. *Reviews in Endocrine & Metabolic Disorders*.

[9] Stevens G. A., Beal T., Mbuya M. N. N. (2022). Micronutrient Deficiencies Among Preschool-Aged Children and Women of Reproductive Age Worldwide: A Pooled Analysis of Individual-Level Data From Population-Representative Surveys. *Lancet Global Health*.

[10] Maurya V. K., Shakya A., Bashir K., Jan K., McClements D. J. (2023). Fortification by Design: A Rational Approach to Designing Vitamin D Delivery Systems for Foods and Beverages. *Comprehensive Reviews in Food Science and Food Safety*.

[11] Fukuda M., Miller D. A., Peppas N. A., McGinity J. W. (2008). Influence of Sulfobutyl Ether Beta-Cyclodextrin (Captisol) on the Dissolution Properties of a Poorly Soluble Drug From Extrudates Prepared by Hot-Melt Extrusion. *International Journal of Pharmaceutics*.

[12] Stella V. J., Rajewski R. A. (2020). Sulfobutylether-β-Cyclodextrin. *International Journal of Pharmaceutics*.

[13] Das S., Subuddhi U. (2015). Studies on the Complexation of Diclofenac Sodium With β-Cyclodextrin: Influence of Method of Preparation. *Journal of Molecular Structure*.

[14] Cid-Samamed A., Rakmai J., Mejuto J. C., Simal-Gandara J., Astray G. (2022). Cyclodextrins Inclusion Complex: Preparation Methods, Analytical Techniques and Food Industry Applications. *Food Chemistry*.

[15] Dahiya M., Saha S., Shahiwala A. (2009). A Review on Mouth Dissolving Films. *Current Drug Delivery*.

[16] Panraksa P., Udomsom S., Rachtanapun P., Chittasupho C., Ruksiriwanich W., Jantrawut P. (2020). Hydroxypropyl Methylcellulose E15: A Hydrophilic Polymer for Fabrication of Orodispersible Film Using Syringe Extrusion 3D Printer. *Polymers*.

[17] Shimizu S., Wada-Hirai A., Li Y., Shimada Y., Otsuka Y., Goto S. (2020). Relationship Between Phase Solubility Diagrams and Crystalline Structures During Dissolution of Cimetidine/Cyclodextrin Complex Crystals. *Journal of Pharmaceutical Sciences*.

[18] Kim S., Thiessen P. A., Bolton E. E. (2016). Pubchem Substance and Compound Databases. *Nucleic Acids Research*.

[19] Dallakyan S., Olson A. J. (2015). Small-Molecule Library Screening by Docking With Pyrx. *Methods in Molecular Biology*.

[20] Rappé A. K., Casewit C. J., Colwell K. S., Goddard W. A., Skiff W. M. (1992). UFF, A Full Periodic Table Force Field for Molecular Mechanics and Molecular Dynamics Simulations. *Journal of the American Chemical Society*.

[21] Trott O., Olson A. J. (2010). Autodock Vina: Improving the Speed and Accuracy of Docking With a New Scoring Function, Efficient Optimization and Multithreading. *Journal of Computational Chemistry*.

[22] Rizvi S. M. D., Shakil S., Haneef M. (2013). A Simple Click by Click Protocol to Perform Docking: Autodock 4.2 Made Easy for Non-Bioinformaticians. *EXCLI Journal*.

[23] Saokham P., Muankaew C., Jansook P., Loftsson T. (2018). Solubility of Cyclodextrins and Drug/Cyclodextrin Complexes. *Molecules*.

[24] Brewster M. E., Loftsson T. (2007). Cyclodextrins as Pharmaceutical Solubilizers. *Advanced Drug Delivery Reviews*.

[25] Khan M. M., Saigal K. S., Shareef S., Sahoo S. C. (2018). Microwave Irradiation: A Green Approach for the Synthesis of Functionalized N-Methyl-1,4-Dihydropyridines. *RSC Advances*.

[26] Rongthong T., Sungthongjeen S., Siepmann J., Pongjanyakul T. (2013). Quaternary Polymethacrylate-Magnesium Aluminum Silicate Films: Molecular Interactions, Mechanical Properties and Tackiness. *International Journal of Pharmaceutics*.

[27] Takeuchi Y., Ikeda N., Tahara K., Takeuchi H. (2020). Mechanical Characteristics of Orally Disintegrating Films: Comparison of Folding Endurance and Tensile Properties. *International Journal of Pharmaceutics*.

[28] Nagaraju T., Gowthami R., Rajashekar M. (2013). Comprehensive Review on Oral Disintegrating Films. *Current Drug Delivery*.

[29] Cupone I. E., Dellera E., Marra F., Giori A. M. (2020). Development and Characterization of an Orodispersible Film for Vitamin D3 Supplementation. *Molecules*.

[30] Abbott K., Starnes E., Collins C. C. (2020). Development of a Robust Dissolution Method for Vitamin D3. https://dc.etsu.edu/asrf/2020/presentations/41/.

[31] Cetindag E., Pentangelo J., Arrieta Cespedes T., Davé R. N. (2020). Effect of Solvents and Cellulosic Polymers on Quality Attributes of Films Loaded With a Poorly Water-Soluble Drug. *Carbohydrate Polymers*.

[32] Delaurent C., Siouffi A. M., Pepe G. (1998). Cyclodextrin Inclusion Complexes With Vitamin D3: Investigations of the Solid Complex Characterization. *Chemia Analityczna*.

[33] Bakirova R., Nukhuly A., Iskineyeva A. (2020). Obtaining and Investigation of the β-Cyclodextrin Inclusion Complex With Vitamin D_3_ Oil Solution. *Scientifica*.

[34] Ebrahimi A., Hamishehkar H., Amjadi S. (2023). Development of Gelatin-Coated Nanoliposomes Loaded With β-Cyclodextrin/Vitamin D3 Inclusion Complex for Nutritional Therapy. *Food Chemistry*.

[35] Liu Y., Chen Y., Gao X., Fu J., Hu L. (2022). Application of Cyclodextrin in Food Industry. *Critical Reviews in Food Science and Nutrition*.

[36] Aiassa V., Garnero C., Zoppi A., Longhi M. R. (2023). Cyclodextrins and Their Derivatives as Drug Stability Modifiers. *Pharmaceuticals*.

[37] Carrier R. L., Miller L. A., Ahmed I. (2007). The Utility of Cyclodextrins for Enhancing Oral Bioavailability. *Journal of Controlled Release*.

[38] Szejtli J., Szente L. (2005). Elimination of Bitter, Disgusting Tastes of Drugs and Foods by Cyclodextrins. *European Journal of Pharmaceutics and Biopharmaceutics*.

[39] Tiwari G., Tiwari R., Rai A. K. (2010). Cyclodextrins in Delivery Systems: Applications. *Journal of Pharmacy and BioAllied Sciences*.

[40] Loftsson T., Brewster M. E., Másson M. (2004). Role of Cyclodextrins in Improving Oral Drug Delivery. *American Journal of Drug Delivery*.

[41] Lockwood S. F., O’Malley S., Mosher G. L. (2003). Improved Aqueous Solubility of Crystalline Astaxanthin (3,3′-Dihydroxy-Beta, Beta-Carotene-4,4′-Dione) by Captisol (Sulfobutyl Ether Beta-Cyclodextrin). *Journal of Pharmaceutical Sciences*.

[42] Tötterman A. M., Schipper N. G. M., Thompson D. O., Mannermaa J. P. (1997). Intestinal Safety of Water-Soluble Beta-Cyclodextrins in Paediatric Oral Solutions of Spironolactone: Effects on Human Intestinal Epithelial Caco-2 Cells. *International Journal of Pharmaceutics*.

[43] Pardeshi C. V., Kothawade R. V., Markad A. R. (2023). Sulfobutylether-β-Cyclodextrin: A Functional Biopolymer for Drug Delivery Applications. *Carbohydrate Polymers*.

[44] Wang R., Zhou H., Siu S. W. I., Gan Y., Wang Y., Ouyang D. (2015). Comparison of Three Molecular Simulation Approaches for Cyclodextrin-Ibuprofen Complexation. *Journal of Nanomaterials*.

[45] Oliveira A. P., Silva A. L. N., Viana L. G. F. C. (2019). β-Cyclodextrin Complex Improves the Bioavailability and Antitumor Potential of Cirsiliol, A Flavone Isolated From *Leonotis nepetifolia* (Lamiaceae). *Heliyon*.

[46] Beig A., Agbaria R., Dahan A. (2015). The Use of Captisol (SBE7-β-CD) in Oral Solubility-Enabling Formulations: Comparison to Hpβcd and the Solubility-Permeability Interplay. *European Journal of Pharmaceutical Sciences*.

[47] Loftsson T., Brewster M. E. (2012). Cyclodextrins as Functional Excipients: Methods to Enhance Complexation Efficiency. *Journal of Pharmaceutical Sciences*.

[48] Al-Heibshy F. N. S., Başaran E., Öztürk N., Demirel M. (2020). Preparation and In Vitro Characterization of Rosuvastatin Calcium Incorporated Methyl Beta Cyclodextrin and Captisol® Inclusion Complexes. *Drug Development and Industrial Pharmacy*.

[49] Albarqi H. A., Alqahtani A. A., Ullah I. (2022). Microwave-Assisted Physically Cross-Linked Chitosan-Sodium Alginate Hydrogel Membrane Doped With Curcumin as a Novel Wound Healing Platform. *AAPS PharmSciTech*.

[50] Das S., Mohanty S., Maharana J., Jena S. R., Nayak J., Subuddhi U. (2020). Microwave-Assisted β-Cyclodextrin/Chrysin Inclusion Complexation: An Economical and Green Strategy for Enhanced Hemocompatibility and Chemosensitivity In Vitro. *Journal of Molecular Liquids*.

[51] Bin Jardan Y. A., Ahad A., Raish M., Al-Mohizea A. M., Al-Jenoobi F. I. (2023). Microwave-Assisted Formation of Ternary Inclusion Complex of Pterostilbene. *Pharmaceuticals*.

[52] Chatzidaki M., Kostopoulou I., Kourtesi C. (2020). β-Cyclodextrin as Carrier of Novel Antioxidants: A Structural and Efficacy Study. *Colloids and Surfaces A: Physicochemical and Engineering Aspects*.

[53] Venuti V., Cannavà C., Cristiano M. C. (2014). A Characterization Study of Resveratrol/Sulfobutyl Ether-β-Cyclodextrin Inclusion Complex and In Vitro Anticancer Activity. *Colloids and Surfaces B: Biointerfaces*.

[54] Majumdar S., Wang X., Sommerfeld S. D. (2018). Cyclodextrin Modulated Type I Collagen Self-Assembly to Engineer Biomimetic Cornea Implants. *Advanced Functional Materials*.

[55] Costa-Júnior E. S., Barbosa-Stancioli E. F., Mansur A. A. P., Vasconcelos W. L., Mansur H. S. (2009). Preparation and Characterization of Chitosan/Poly(Vinyl Alcohol) Chemically Crosslinked Blends for Biomedical Applications. *Carbohydrate Polymers*.

[56] Tripathi S., Mehrotra G. K., Dutta P. K. (2009). Physicochemical and Bioactivity of Cross-Linked Chitosan-PVA Film for Food Packaging Applications. *International Journal of Biological Macromolecules*.

[57] Gazquez-Navarro J. J., Garcia-Sanoguera D., Balart R., Garcia-Garcia D., Gomez-Caturla J. (2024). A New Use of Polysorbate-Type Nonionic Surfactants as Plasticizers for Highly Flexible Poly(Lactide) Formulations. *Journal of Polymers and the Environment*.

[58] El-Feky Y. A., Mostafa D. A., Al-Sawahli M. M. (2020). Reduction of Intraocular Pressure Using Timolol Orally Dissolving Strips in the Treatment of Induced Primary Open-Angle Glaucoma in Rabbits. *Journal of Pharmacy and Pharmacology*.

[59] Nair A. B., Kumria R., Harsha S., Attimarad M., Al-Dhubiab B. E., Alhaider I. A. (2013). In Vitro Techniques to Evaluate Buccal Films. *Journal of Controlled Release*.

[60] Pawar R., Sharma R., Sharma P., Darwhekar G. N. (2019). A Review on Mouth Dissolving Film. *Journal of Drug Delivery and Therapeutics*.

[61] Zhao Y. H., Guo Y. Z., Wei Q. (2009). Influence of Specimen Dimensions and Strain Measurement Methods on Tensile Stress-Strain Curves. *Materials Science and Engineering: A*.

[62] Baliga S., Muglikar S., Kale R. (2013). Salivary pH: A Diagnostic Biomarker. *Journal of Indian Society of Periodontology*.

[63] FDA (2008). Orally Disintegrating Tablets. https://www.fda.gov/regulatory-information/search-fda-guidance-documents/orally-disintegrating-tablets.

[64] Maurya V. K., Aggarwal M. (2017). Factors Influencing the Absorption of Vitamin D in GIT: An Overview. *Journal of Pharmacy and Pharmacology*.

[65] Ita K. (2025). Chapter 15-Buccal and Sublingual Drug Delivery Systems. *Drug Delivery*.

[66] Shahiwala A., Misra A., Shahiwala A. (2021). Chapter 2-Applications of Polymers in Buccal Drug Delivery. *Applications of Polymers in Drug Delivery*.

[67] Şenel S., Hincal A. A. (2001). Drug Permeation Enhancement via Buccal Route: Possibilities and Limitations. *Journal of Controlled Release*.

[68] Sabra R., Kirby D., Chouk V., Malgorzata K., Mohammed A. R. (2024). Buccal Absorption of Biopharmaceutics Classification System III Drugs: Formulation Approaches and Mechanistic Insights. *Pharmaceutics*.

